# Electroacupuncture improves depression with constipation by balancing gut microbiota in WKY rats

**DOI:** 10.3389/fmicb.2025.1680596

**Published:** 2026-01-28

**Authors:** Xiang Li, Guancheng Li, Kaiyu Cui, Xuan Yin, Wei Yang, Wei Li, Shifen Xu

**Affiliations:** Shanghai Municipal Hospital of Traditional Chinese Medicine Affiliated to Shanghai University of Traditional Chinese Medicine, Shanghai, China

**Keywords:** depression, fecal microbiota transplantation, 16S sequencing, gut microbiota, electroacupuncture

## Abstract

Accumulating evidence underscores the pivotal role of the gut microbiota in the pathogenesis of depression. In this study, we employed the Wistar-Kyoto (WKY) rat, a well-established animal model of depression comorbid with constipation. Using 16S rRNA gene sequencing, we characterized the gut microbial community structure and investigated the impact of microbiota modulation on depressive-like behaviors and gastrointestinal dysfunction. Comparative analyses revealed that WKY rats exhibited significantly increased relative abundances of Proteobacteria, Bacteroidetes, and Desulfobacterota, accompanied by a marked reduction in Firmicutes compared to control Wistar rats. Fecal microbiota transplantation (FMT) demonstrated that colonization of WKY rats with microbiota from Wistar rats restored microbial composition, improved depressive-like behaviors, and normalized gut motility. In contrast, Wistar rats receiving microbiota from WKY donors developed depression-like phenotypes and impaired intestinal function. Moreover, electroacupuncture (EA) treatment not only alleviated depressive-like behaviors in WKY rats but also promoted recovery of colonic epithelial ultrastructure and rebalanced gut microbial composition. Collectively, these findings demonstrate that both FMT and EA effectively ameliorate depressive behaviors and constipation in WKY rats, with EA likely exerting its therapeutic effects through modulation of the gut microbiota.

## 1 Introduction

Depression is a common affective disorder marked by persistent and significant low mood, affecting approximately 16% of the global population. According to the World Health Organization (WHO), depression is currently the fourth leading contributor to the global disease burden and is projected to become the leading cause of disability by 2030 (Muñoz and Bunge, 2016). This underscores the urgent need to elucidate the pathogenesis of depression and to develop novel therapeutic strategies. Wistar Kyoto (WKY) rats have been widely established as a model for endogenous depression, owing to their characteristic behavioral traits such as social withdrawal, reduced locomotor activity, decreased body weight, and disrupted circadian rhythms (Aleksandrova et al., 2019; Redei et al., 2023).

The gut microbiota, the largest microbial ecosystem in the host, plays a significant role in regulating central nervous system functions and mediating the pathogenesis of depression through multiple pathways along the gut-brain axis, including metabolic, immune, and vagus nerve signaling. Emerging evidence highlights the bidirectional nature of microbial signaling, which may simultaneously modulate both cerebral and gastrointestinal functions (Wang et al., 2023). While increasing research has focused on alterations in the gut microbiota structure in WKY rats exhibiting depressive phenotypes (Sun et al., 2019; Dash et al., 2015), the causal relationship between these microbial changes and depressive behaviors remains insufficiently explored.

Fecal microbiota transplantation (FMT), an emerging therapeutic approach, has shown potential in depression intervention research. Clinical evidence demonstrates that FMT can alleviate depressive symptoms, often accompanied by restoration of the gut microbiome (Jiang et al., 2024). Mechanistic studies reveal that FMT ameliorates depression through microbiota-mediated regulation of inflammatory pathways (Liu et al., 2024). Notably, preclinical studies have shown that transplantation of healthy donor microbiota significantly improves depression-like behaviors in rodent models (Wang et al., 2024), indicating that a healthy gut microbiota can reverse depressive pathophysiology. These findings highlight the dual significance of FMT, both as a therapeutic modality and as an experimental tool for investigating the role of the microbiota in depression.

As a traditional Chinese therapeutic intervention, acupuncture has demonstrated distinct advantages in the management of depression (Li et al., 2015). Our research team has accumulated clinical evidence indicating that acupuncture significantly reduces scores on the Hamilton Depression Rating Scale (HAMD) and the Self-Rating Anxiety Scale (SAS), while also improving outcomes on the Pittsburgh Sleep Quality Index (PSQI) (Yin et al., 2022; Li et al., 2020). Building on this clinical foundation, we have shown in WKY rats that EA ameliorates depressive-like behaviors by regulating hippocampal metabolism and repairing synaptic ultrastructure, particularly by enhancing synaptic transmission efficiency in the dorsal raphe nucleus (Zeng et al., 2025; Han et al., 2018). However, whether EA modulates gut microbiota composition to exert its antidepressant effects in WKY rats remains to be elucidated.

In this study, we employed 16S rRNA sequencing to characterize differences in gut microbiota composition between WKY rats and Wistar controls. To establish the direct regulatory role of the gut microbiota in depressive-like behaviors, bidirectional FMT was conducted. We further assessed the effects of EA on both behavioral outcomes and gut microbial composition in WKY rats. Additionally, antibiotic-induced microbiota depletion was used to determine the extent to which gut microbiota contributes to the therapeutic efficacy of EA. This integrated approach—encompassing microbial profiling, fecal transplantation, and targeted microbial disruption—provides a comprehensive framework for investigating gut–brain interactions in the pathophysiology and treatment of depression.

## 2 Materials and methods

### 2.1 Animals

Male specific pathogen-free (SPF) grade Wistar and Wistar Kyoto (WKY) rats, approximately 6 weeks old, were obtained from Beijing Vital River Laboratory Animal Technology Co., Ltd. All experimental procedures were conducted in accordance with the ethical guidelines of Shanghai University of Traditional Chinese Medicine and were approved under protocol number PZSHUTCM200821009. Upon arrival at the animal facility, rats were acclimated for 1 week with *ad libitum* access to food and water. Environmental conditions in the animal room were maintained at a temperature of 22–27 °C, relative humidity of 40–60%, and noise levels below 50 dB, under a controlled light-dark cycle.

### 2.2 Intervention methods

EA Group: Rats in the depression model group received EA treatment. Acupoints used were Baihui (GV20) and Zusanli (ST36), identified according to the “Atlas of Acupoints for Experimental Animals.” For the procedure, Baihui was obliquely inserted 5 mm anteriorly, while Zusanli was vertically inserted to a depth of 3 mm. A Huatuo EA instrument was used, with the positive electrode connected to Baihui and the negative electrode connected to Zusanli. Continuous wave stimulation was applied at a frequency of 2 Hz (Han et al., 2018; She et al., 2015), with the current adjusted according to the rats' tolerance, ensuring that the head trembled without vocalization. The needles were retained for 15 min, once per day, for three consecutive weeks. To avoid stress-induced interference with the experimental results due to animal restraint, the rats were placed in specially designed plastic cages during EA, allowing some degree of movement.

Sham EA Group: Rats in the depression model group received a placebo sham EA intervention. Following an internationally recognized sham acupuncture research protocol (Lao et al., 2004), the same handling procedures as in the EA group were applied. However, the acupuncture needles were fixed superficially at the Baihui and Zusanli points without skin penetration, thereby minimizing non-specific needle effects. Electrodes were attached to the needles but without current, with the intervention time matching that of the EA group. The feasibility of this sham procedure was confirmed in preliminary experiments. Following the final EA session, fecal samples were collected from the EA group only; no fecal samples were obtained from the sham EA group. Behavioral testing and gastrointestinal motility assessments were conducted beginning the next day.

### 2.3 Behavioral testing

#### 2.3.1 Open field test

A square open field apparatus measuring 50 cm × 50 cm, with 40 cm-high black walls on all sides, was used to assess exploratory behavior and locomotor activity in rats. Prior to each trial, the open field was cleaned with 75% ethanol and allowed to air dry completely. Each rat was then placed individually into the center of the arena, and its behavior was recorded for a 5-min session using a behavioral tracking platform. The floor of the open field was divided into nine equal squares, with the central square (area of 125 cm^2^) designated as the central zone. The total distance traveled by the rats in the central zone was recorded as the central distance, the total time spent in the central zone as the central time, and the total number of rearing behaviors (forelimbs off the ground, hindlimbs on the ground) as the rearing count. Total locomotor activity was assessed by measuring the total distance traveled throughout the arena. Behavioral data were analyzed using the Shanghai Xinruan behavioral analysis system.

#### 2.3.2 Novelty-suppressed feeding test

A test box measuring 50 cm × 50 cm × 40 cm was used, with a 2 cm-thick layer of wood shavings covering the floor. A small sugar pellet was placed at the center of the box on a piece of white paper. Prior to the test, rats were food-deprived for 48 h to standardize motivation for food-seeking behavior. During the test, each rat was placed into the box from a randomly selected corner, and its behavior was recorded for 5 min using a video monitoring system. The latency to approach and begin eating the sugar pellet—defined as the time from placement in the box to the point at which the rat grasped and began consuming the pellet with its forelimbs—was measured.

#### 2.3.3 Forced swim test

A glass cylinder (height: 45 cm; diameter: 18 cm) was filled with water to a depth of 30 cm, maintained at a temperature of 24–28 °C. The rats were placed in the water, and software was used to record the immobility time within 5 min.

### 2.4 Gastrointestinal motility assessment

#### 2.4.1 Body weight change measurement

The rats' body weight was first recorded upon entry into the SPF animal facility and designated as M1. Prior to the initiation of behavioral testing, body weight was measured again and recorded as M2. The change in body weight was calculated as M0 = M2 – M1, representing the net weight change during the acclimation and intervention period.

#### 2.4.2 Intestinal transit time

The rats were allowed free access to food and water prior to the test. A 6% carmine red solution was prepared in 0.5% methylcellulose and administered via oral gavage at a dose of 1 mL/100 g body weight. The time of gavage completion was recorded for each rat. Following administration, rats were monitored continuously, and the latency to the first appearance of red-colored feces was recorded. This latency served as an indicator of gastrointestinal transit time.

#### 2.4.3 Fecal water content measurement

Fecal pellets were collected from each rat over a 24-h period and weighed immediately (W0). The weighed feces were then dried in a far-infrared rapid thermostatic drying oven at 60 °C. The feces were reweighed at 30-min intervals a constant weight was achieved (W1). The fecal water content was calculated as: (W0 - W1)/W0.

### 2.5 Fecal microbiota transplantation

WKY rats were divided into two groups: one group received phosphate-buffered saline (PBS) oral gavage (WKY + PBS), while the other group received FMT from Wistar rats (WKY+FMT). Similarly, Wistar rats were divided into two groups: one group received PBS oral gavage (Wistar + PBS), while the other group received FMT from WKY rats (Wistar + FMT). Fresh feces were collected daily via gentle abdominal massage and immediately placed into sterile cryogenic tubes. Fecal samples from rats within the same group were pooled, weighed, and diluted with sterile PBS at a ratio of 1 g feces to 10 mL PBS. The mixture was homogenized using a sterile pestle and centrifuged at 1,200 × g for 5 min. The resulting supernatant was collected and transferred into new sterile centrifuge tubes for oral gavage. Prior to the start of the FMT procedure, recipient rats were housed in sterile isolation cages for 1 week to allow environmental adaptation. FMT was initiated on the eighth day, with rats in the WKY+FMT and Wistar+FMT groups receiving a daily gavage of 1 mL/100 g body weight of donor fecal supernatant for seven consecutive days. Control groups received an equivalent volume of sterile PBS.

### 2.6 Sample preparation

Following completion of all behavioral assessments, fecal samples were collected from the rats for microbiota analysis. The perianal area of each rat was disinfected using an alcohol swab to minimize contamination. Feces were collected directly into sterile EP tubes, ensuring a minimum of three pellets per rat. Samples from ten rats per group were selected for downstream analysis. Collected samples were immediately flash-frozen in liquid nitrogen and subsequently transferred to a −80 °C freezer for long-term storage. All fecal samples were preserved under sterile conditions and later used for 16S rDNA sequencing to assess gut microbiota composition.

### 2.7 ELISA analysis

The concentrations of Ghrelin and vasoactive intestinal polypeptide (VIP) were measured using ELISA kits (Hangzhou Lianke Biology Technology Co., Ltd.), and the concentration of Interleukin-1 beta (IL-1β), Tumor Necrosis Factor-alpha (TNF-α), Interleukin-6 (IL-6) was measured using an ELISA kit (Hangzhou Lianke Biology Technology Co., Ltd., EK306) following the manufacturer's instructions. The concentrations of the target proteins were determined using a standard protein curve.

### 2.8 DNA extraction, PCR amplification, and sequencing of fecal samples

All fecal samples were frozen at −80 °C before DNA extraction and sequencing analysis. The subsequent procedures were performed by Majorbio Bio-Pharm Technology Co., Ltd. (Shanghai, China). Microbial DNA was extracted from fecal samples using the E.Z.N.A.^®^ soil DNA kit according to the manufacturer's protocol. DNA concentration and purity were determined using a NanoDrop 2000 UV-Vis spectrophotometer, and DNA quality was assessed by 1% agarose gel electrophoresis. The V3–V4 region of the bacterial 16S rRNA gene was targeted for amplification using primers 338F (5′-ACTCCTACGGGAGGCAGCAG-3′) and 806R (5′-GGACTACHVGGGTWTCTAAT-3′) on a thermal cycler PCR system (GeneAmp 9700, ABI, USA). The PCR conditions were as follows: initial denaturation at 95 °C for 3 min; followed by 30 cycles at 27 °C, each cycle consisting of 95 s; annealing at 30 °C for 55 s; extension at 45 °C for 72 s; and a final extension at 72 °C for 10 min. The PCR products were extracted from a 2% agarose gel, further purified using the AxyPrep DNA Gel Extraction Kit, and quantified using a Quantus™ Fluorometer according to the manufacturer's protocol.

### 2.9 Sequencing data processing

Raw FASTQ files were demultiplexed based on barcode and primer sequences. Quality control of raw reads was performed using Trimmomatic to remove low-quality bases and sequencing adapters. Subsequently, paired-end reads were merged using FLASH. The resulting sequences were clustered into operational taxonomic units (OTUs) at 97% sequence similarity using the UPARSE algorithm, during which chimeric sequences were identified and eliminated. Taxonomic classification of representative OTU sequences was performed using the Ribosomal Database Project (RDP) classifier against the SILVA 16S rRNA database (version 132 for bacteria), applying a minimum confidence threshold of 70%. All bioinformatics analyses were conducted using the online platform provided by Majorbio Bio-Pharm Technology Co., Ltd. (Shanghai, China).

## 3 Statistical analysis

All experimental data were analyzed and visualized using GraphPad Prism 8.0. Data are presented as mean ± standard deviation (Mean ± SD). Prior to statistical testing, data were assessed for normality and homogeneity of variance. For data that met the assumptions of normal distribution and equal variance, intergroup comparisons were conducted using the *t*-test. When data did not satisfy these assumptions, the Mann-Whitney U test was applied as a non-parametric alternative. For multiple group comparisons of gut microbiota, the Kruskal-Wallis rank-sum test and *post-hoc* tests were employed. Spearman correlation analysis was used to assess the relationship between gut microbiota and behavior. *P* < 0.05 was considered statistically significant.

The sample size was determined a priori using G^*^Power 3.1 software (Heinrich-Heine-Universität Düsseldorf, Germany). Based on a one-way ANOVA design with four groups (as in our FMT and behavior experiments), we set the following parameters: Effect size *f* = 0.5,α = 0.05 (Type I error rate), Power (1 – β) = 0.70, The calculated minimum total sample size was 40, i.e., 10 animals per group. For two-group comparisons (e.g., ELISA assays, behavioral tests), we conducted a two-tailed independent-samples *t*-test power analysis: Effect size *d* = 0.95, α = 0.05, Power = 0.70, This yielded a minimum sample size of 15 animals per group.

## 4 Results

### 4.1 WKY rats exhibit depression-like behavior and impaired gastrointestinal function

**Figure 1:**
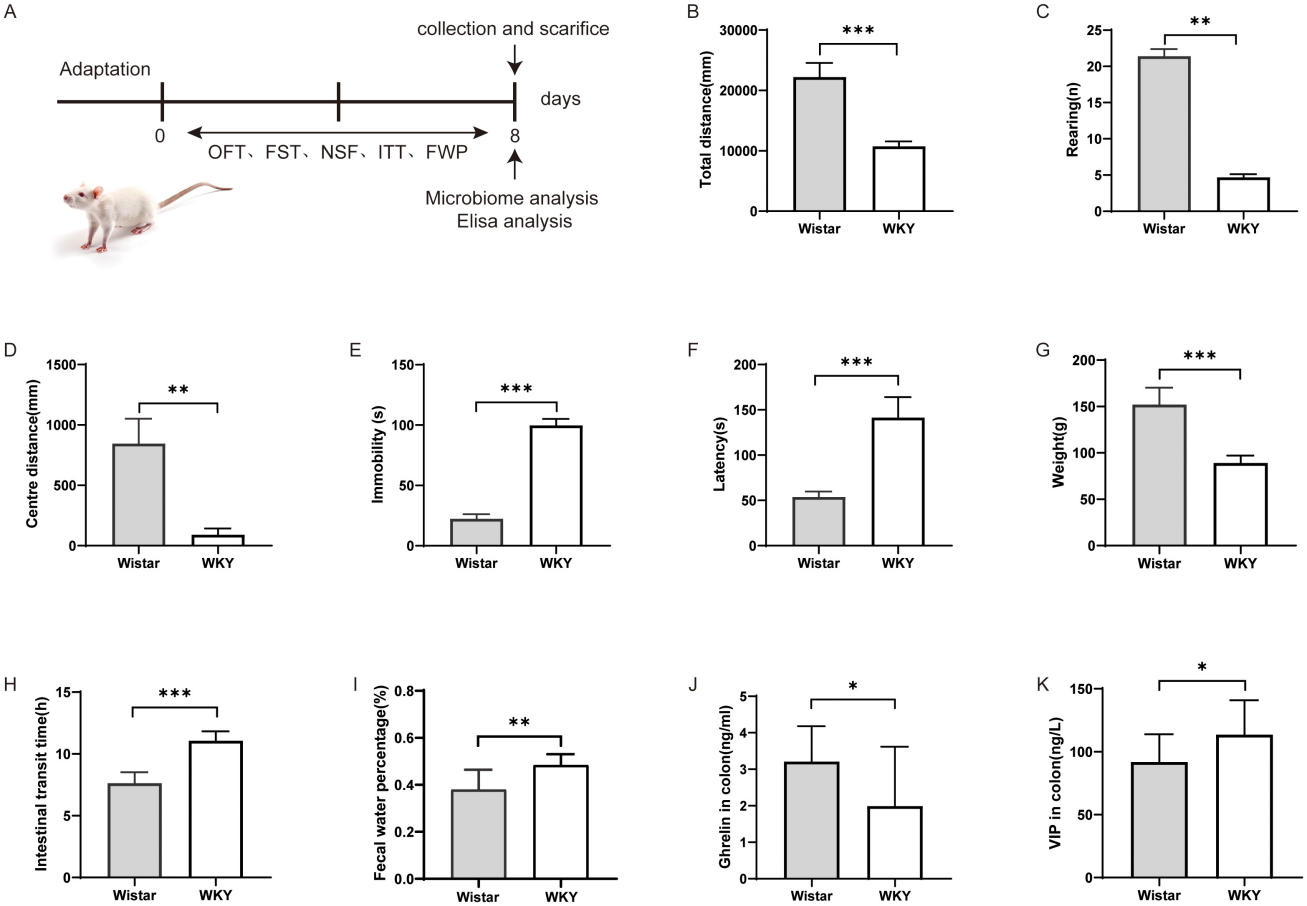
Changes in depression-like behavior, body weight, and gastrointestinal function in Wistar and WKY rats. **(A)** Schematic timeline of the experimental design. **(B)** Total moving distance in the open field test. **(C)** Number of rearing events in the open field test. **(D)** Central activity distance in the open field test. **(E)** Immobility time in the forced swimming test. **(F)** Feeding latency in the novelty-suppressed feeding test. **(G)** Changes in body weight. **(H)** Intestinal transit time. **(I)** Fecal water content. **(J)** Concentration of ghrelin in the colon. **(K)** Concentration of vasoactive intestinal peptide (VIP) in the colon. (Wistar, *n* = 15, WKY, *n* = 15, Mean ± SD, *P < 0.05, **P < 0.01, ***P < 0.001).

Baseline behavioral comparisons between WKY rats and Wistar rats revealed significant differences across multiple domains. In the open field test (Figures 1A–D), WKY rats demonstrated a significantly reduced total distance traveled (*t* = 4.584, *df* = 28, *P* < 0.001), fewer rearing behaviors (*t* = 15.49, *df* = 28, *P* < 0.01), and a decreased central zone distance (*t* = 3.546, *df* = 28, *P* < 0.01), indicating impaired exploratory behavior and increased anxiety-like behavior. In the forced swim test (Figure 1E), WKY rats exhibited significantly prolonged immobility time (*t* = 11.60, *df* = 28, *P* < 0.001), while in the novelty-suppressed feeding test (Figure 1F), they showed an increased latency to feed (*t* = 14.43, *df* = 28, *P* < 0.001), both indicative of depressive-like behavior. Gastrointestinal function assessments (Figures 1G–I) further revealed that, compared to Wistar rats, WKY rats experienced notable weight loss (*t* = 12.31, *df* = 28, *P* < 0.001), prolonged intestinal transit time (*t* = 11.32, *df* = 28, *P* < 0.001), and an elevated fecal water content percentage (*t* = 4.197, *df* = 28, *P* < 0.01). These findings collectively indicate that WKY rats display both depressive-like behaviors and compromised gastrointestinal function, supporting their use as a potential animal model for comorbid depression and constipation. To further elucidate the mechanisms underlying gastrointestinal dysfunction in these rats, we measured colonic levels of ghrelin and vasoactive intestinal peptide (VIP), which are known to be positively and negatively associated with gastrointestinal motility, respectively. ELISA analysis showed that Ghrelin levels were significantly lower in WKY rats compared to Wistar rats (*t* = 2.232, *df* = 22, *P* < 0.05), whereas VIP levels were elevated in WKY rats compared to Wistar rats (*t* = 2.139, *df* = 22, *P* < 0.05) (Figures 1J, K). These data suggest that depressed rats exhibit significant constipation symptoms, consistent with our behavioral findings.

### 4.2 Changes in gut microbiota composition in WKY Rats

**Figure 2:**
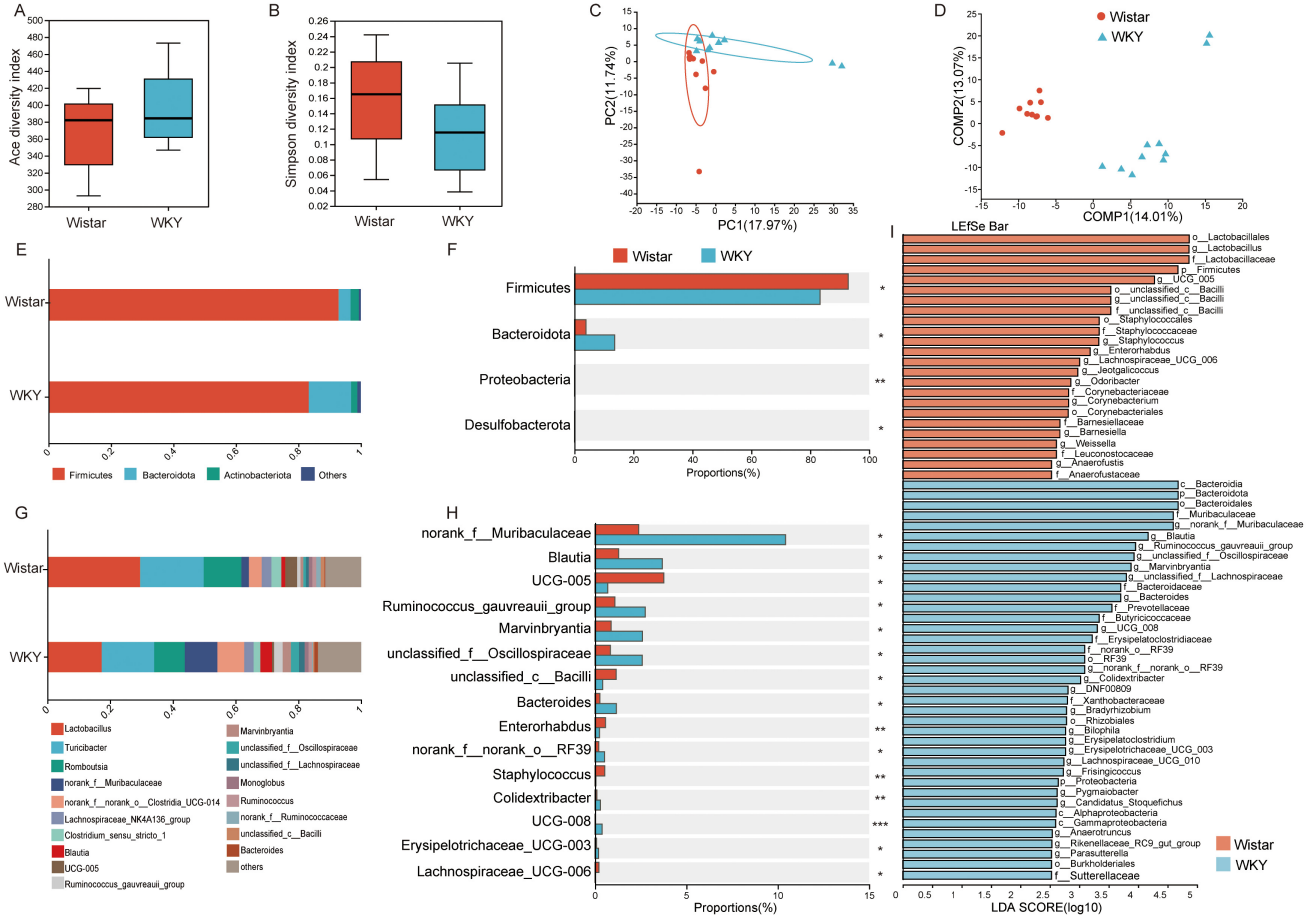
Changes in gut microbiota composition in depressed rats. **(A)** Ace index of α-diversity. **(B)** Simpson index of α-diversity. **(C)** PCA analysis of beta diversity. **(D)** PLS-DA analysis. **(E)** Composition of gut microbiota at the phylum level in both groups. **(F)** Differential abundance analysis of gut microbiota at the phylum level between the two groups. **(G)** Composition of gut microbiota at the genus level in both groups. **(H)** Differential abundance analysis of gut microbiota at the genus level between the two groups. **(I)** Linear discriminant analysis (LDA) of gut microbiota between the two groups. (Wistar, *n* = 8, WKY, *n* = 8, Mean ± SD, *P < 0.05, **P < 0.01, ***P < 0.001).

To investigate differences in gut microbiota composition between Wistar and WKY rats, 16S rRNA gene sequencing was performed. Alpha diversity analysis showed no significant differences in species richness or diversity between the two groups (Figures 2A, B), indicating a comparable overall microbial community structure. However, beta diversity analysis revealed distinct compositional differences. Principal coordinate analysis (PCA) demonstrated that samples clustered primarily by group, reflecting high intra-group similarity and inter-group divergence (Figure 2C). Moreover, Partial Least Squares Discriminant Analysis (PLS-DA) showed a clear separation between the microbial profiles of the two groups (Figure 2D), further supporting the presence of significant differences in gut microbiota composition between Wistar and WKY rats.

At the phylum level (Figures 2E, F), compared to Wistar rats, WKY rats exhibited a significant increase in the relative abundance of Proteobacteria, Bacteroidota, and Desulfobacterota, whereas the relative abundance of Firmicutes was significantly decreased. At the genus level (Figures 2G, H), a comparison of relative abundances between the two groups showed that norank_f__Muribaculaceae, Blautia, Ruminococcus gauvreauii group, Marvinbryantia, unclassified_f__Oscillospiraceae, Bacteroides, norank_f__norank_o__RF39, Colidextribacter, UCG-008, and Erysipelotrichaceae_UCG-003 were significantly higher in WKY rats compared to the control group, whereas UCG-005, unclassified_c__Bacilli, Enterorhabdus, Staphylococcus, and Lachnospiraceae_UCG-006 were significantly higher in Wistar rats than in WKY rats.

Building on the observed compositional differences, dominant microbial taxa distinguishing the two groups were identified using Linear Discriminant Analysis Effect Size (LEfSe) (Figure 2I). With an LDA score threshold of ≥2.5, taxa with a relatively strong influence at various taxonomic levels—from phylum to genus—were determined. It was found that Bacteroidota (LDA = 4.674), Bacteroidia (LDA = 4.674), Bacteroides (LDA = 4.174), Muribaculaceae (LDA = 4.592), and norank_f__Muribaculaceae (LDA = 4.592) were significantly enriched in WKY rats and played a key distinguishing role, whereas Lactobacillaceae (LDA = 5.470), Lactobacillus (LDA = 5.470), Firmicutes (LDA = 5.968), and UCG_005 (LDA = 4.576) were significantly enriched in Wistar rats. These results suggest that microbial alterations in WKY rats are primarily characterized by an increased relative abundance of taxa within the phylum Bacteroidota, which may be associated with depressive-like behaviors and constipation symptoms.

### 4.3 FMT alleviates gut microbiota dysbiosis in depressed rat models

**Figure 3:**
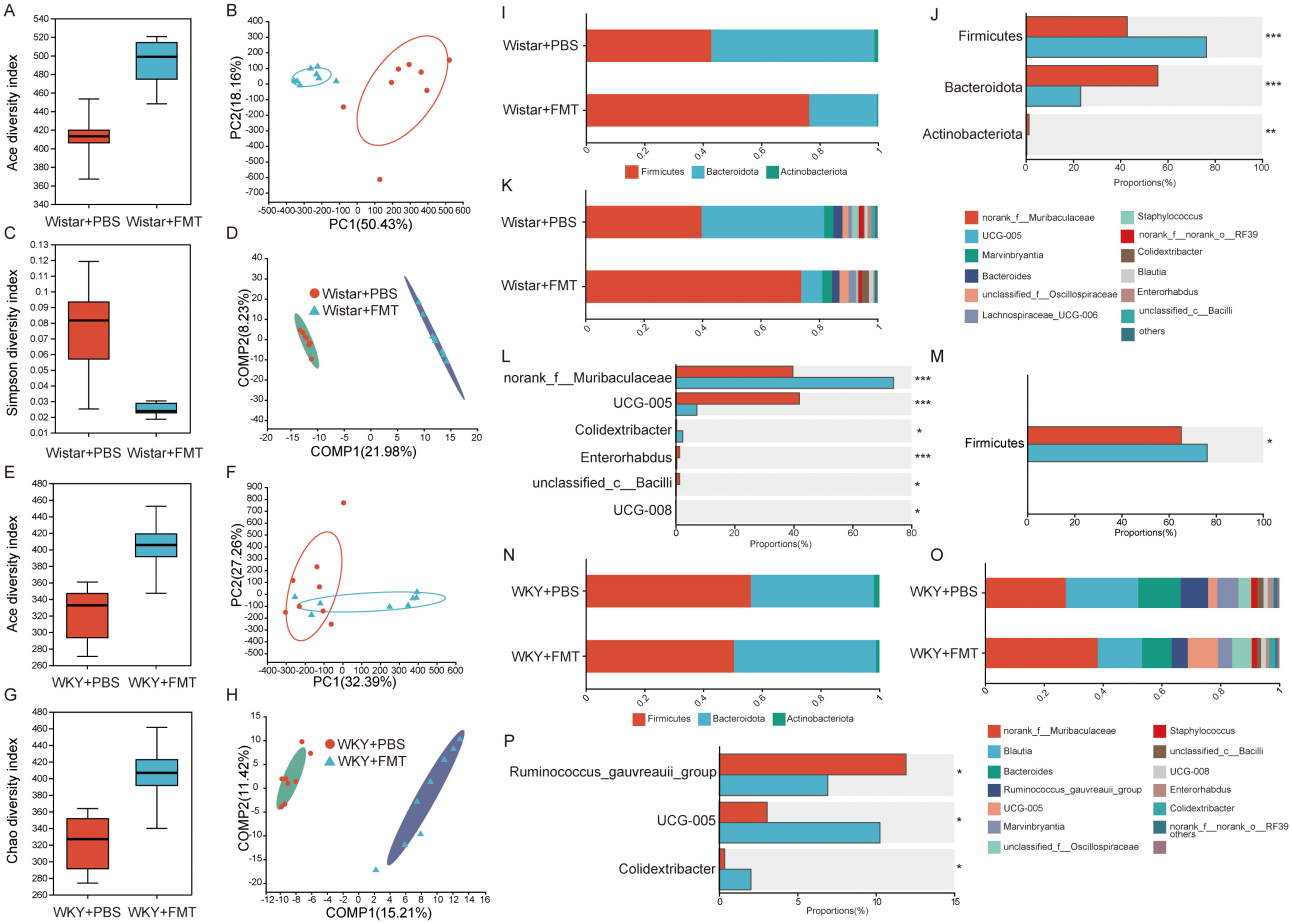
Changes in gut microbiota across groups after fecal microbiota transplantation. **(A)** Ace index of α-diversity in Wistar + PBS and Wistar + FMT groups. **(B)** PCA analysis in Wistar + PBS and Wistar + FMT groups. **(C)** Simpson index of α-diversity in Wistar + PBS and Wistar + FMT groups. **(D)** PLS-DA analysis in Wistar + PBS and Wistar + FMT groups. **(E)** Ace index of α-diversity in WKY+PBS and WKY+FMT groups. **(F)** PCA analysis in WKY + PBS and WKY + FMT groups. **(G)** Chao index of α-diversity in WKY + PBS and WKY + FMT groups. **(H)** PLS-DA analysis in WKY + PBS and WKY + FMT groups. **(I)** Composition of intestinal flora at phylum level in Wistar + PBS and Wistar + FMT groups. **(J)** Differential analysis of gut flora in Wistar + PBS and Wistar + FMT groups at the phylum level. **(K)** Composition of intestinal flora at genus level in Wistar + PBS and Wistar + FMT groups. **(L)** Differential analysis of gut flora in Wistar + PBS and Wistar + FMT groups at the genus level. **(M)** Differential analysis of gut flora in WKY + PBS and WKY + FMT groups at the phylum level. **(N)** Composition of intestinal flora at the phylum level in WKY + PBS and WKY + FMT groups. **(O)** Composition of intestinal flora at the genus level in WKY + PBS and WKY + FMT groups. **(P)** Differential analysis of gut flora in WKY + PBS and WKY + FMT groups at the genus level. (Wistar + PBS, *n* = 10, Wistar + FMT, *n* = 10, WKY + PBS, *n* = 10, WKY + FMT, *n* = 10. T test was used for comparison between the two groups. *P < 0.05, **P < 0.01, ***P < 0.001).

To investigate the causal relationship between alterations in gut microbiota and depressive-like behavior, fecal microbiota transplantation (FMT) experiments were conducted. Following a 1-week intervention, 16S rRNA sequencing was performed on rats from each group. In the first experiment, fecal microbiota from WKY rats were transplanted into Wistar rats. As shown in Figures 3A–D, alpha diversity analysis revealed no significant differences in ACE and Simpson indices between the Wistar + FMT and Wistar + PBS groups, although the ACE index was slightly higher in the Wistar + FMT group, suggesting increased species richness. Beta diversity analysis based on weighted Bray-Curtis distances indicated notable differences in microbiota composition between the two groups, with PLS-DA both demonstrating clear separation, thereby confirming structural alterations in gut microbial communities. To further determine whether these FMT-induced changes mirrored the differential taxa previously identified, we compared the microbial composition at both the phylum and genus levels (Figures 3I–L). At the phylum level, the Wistar + FMT group exhibited significantly reduced relative abundances of Firmicutes (*P* < 0.001) and Actinobacteriota (*P* < 0.01), and significantly increased abundances of Bacteroidota (*P* < 0.001) and Patescibacteria (*P* < 0.05), relative to the Wistar + PBS group. At the genus level, the Wistar + FMT group showed increased abundances of norank_f__Muribaculaceae, Colidextribacter, and UCG-008, and decreased abundances of UCG-005, Enterorhabdus, and unclassified_c__Bacilli. These shifts were consistent with the microbial signatures observed in WKY rats (Figures 3E–H).

In a reciprocal experiment, fecal microbiota from Wistar rats were transplanted into WKY rats to determine whether gut microbiota from behaviorally normal rats could modulate the microbial profile of depressed rats. Alpha diversity analysis revealed no significant differences in the ACE and Simpson indices between the WKY + PBS and WKY + FMT groups, indicating similar species richness and diversity. However, beta diversity analysis showed clear separation in microbial community structure between the two groups, as confirmed by both PLS-DA, suggesting that FMT led to distinct compositional changes in the gut microbiota of WKY rats (Figures 3M–P). At the phylum level, only the relative abundance of Firmicutes was significantly increased in the WKY + FMT group compared to the WKY+PBS group (*P* < 0.05), whereas no significant differences were observed for other phyla. At the genus level, the WKY+FMT group exhibited increased relative abundances of UCG-005, unclassified_c__Bacilli, Enterorhabdus, Staphylococcus, and Lachnospiraceae_UCG-006, whereas the relative abundances of norank_f__Muribaculaceae, Blautia, Ruminococcus gauvreauii group, Marvinbryantia, unclassified_f__Oscillospiraceae, Bacteroides, norank_f__norank_o__RF39, Colidextribacter, UCG-008, and Erysipelotrichaceae_UCG-003 were lower. These findings suggest that transplantation of gut microbiota from Wistar rats partially reshaped the microbial community in WKY rats, making it more similar to that of normal rats. Thus, a gut microbiota structure resembling that of behaviorally healthy animals was successfully established in WKY rats through FMT.

**Figure 4:**
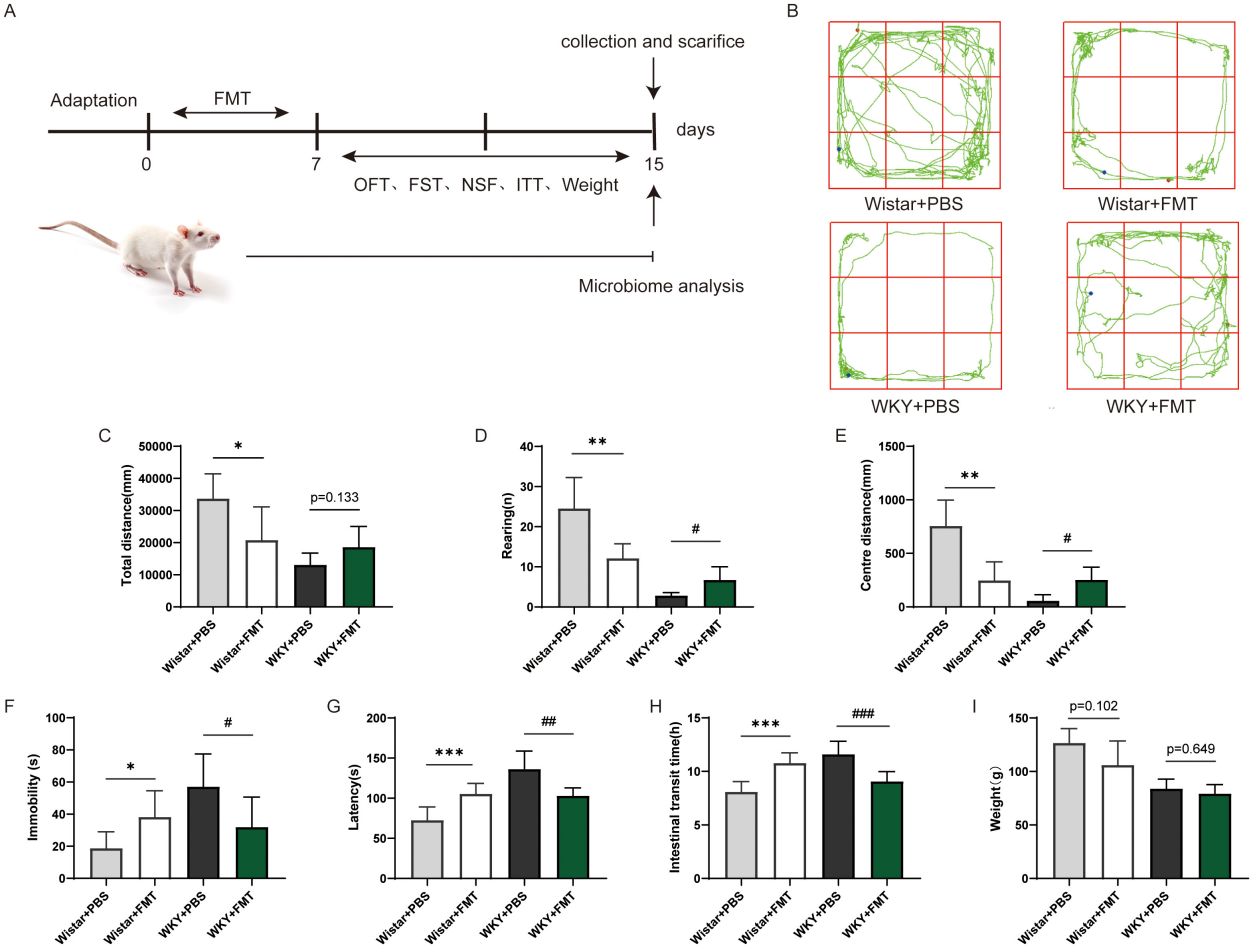
Depression-like behavior and changes in gastrointestinal function after FMT. **(A)** Experimental timeline for fecal microbiota transplantation. **(B)** Representative motion trajectories of Wistar and WKY rats following FMT. **(C)** Total moving distance in the open field test. **(D)** Number of rearing events in the open field test. **(E)** Central activity distance in the open field test. **(F)** Immobility time in the forced swimming test. **(G)** Feeding latency in the novelty suppressed feeding test. **(H)** Gastrointestinal transit time across the four groups. **(I)** Body weight changes across the four groups (Wistar+PBS, *n* = 10, Wistar + FMT, *n* = 10, WKY + PBS, *n* = 10, WKY + FMT, *n* = 10, Mean ± SD). Wistar + PBS vs. Wistar + FMT *P < 0.05, **P < 0.01, ***P < 0.001; WKY + PBS vs. WKY + FMT.^#^*P* < 0.05, ^##^*P* < 0.01, ^###^*P* < 0.001.

To assess the behavioral consequences of FMT, we conducted a series of behavioral tests on the four groups of rats. In the open field test (Figures 4A–E), the Wistar+FMT group exhibited significant reductions in total distance traveled (*F* = 3.162, *df* = 16.67, *P* < 0.05), rearing times (*F* = 4.574, *df* = 13.83, *P* < 0.01), and central distance (*F* = 1.710, *df* = 16.30, *P* < 0.01) compared to the Wistar + PBS group. In the forced swim test (Figure 4F), immobility time was significantly increased in the Wistar + FMT group (*F* = 3.161, *df* = 15.20, *P* < 0.05). Likewise, in the novelty-suppressed feeding test (Figure 4G), latency to feed was markedly prolonged in the Wistar + FMT group (*F* = 4.880, *df* = 17.06, *P* < 0.001). Conversely, FMT from Wistar rats to WKY rats attenuated depressive behaviors. Although the total distance in the open field test did not differ significantly between the WKY + FMT and WKY + PBS groups (*F* = 2.347, *df* = 14.33, *P* = 0.1331), the WKY + FMT group exhibited significantly increased rearing frequency (*F* = 3.633, *df* = 10.02, *P* < 0.05) and central distance (*F* = 1.480, *df* = 12.94, *P* < 0.01). Immobility time in the forced swim test was reduced in the WKY+FMT group (*F* = 2.865, *df* = 17.88, *P* < 0.05), and latency to feed in the novelty-suppressed feeding test was also decreased (*F* = 4.219, *df* = 12.40, *P* < 0.01), compared to the WKY + PBS group. Together, these findings demonstrate that transplantation of gut microbiota from depressive rats induced depressive phenotypes in healthy rats, while transplantation from healthy rats alleviated depressive symptoms in depressive rats. This provides strong evidence for a causal role of gut microbiota in modulating depression-like behaviors.

### 4.4 Correlation between depressive behaviors and gut microbiota in rats

The association between differentially abundant gut microbiota and behavioral outcomes was evaluated using Spearman correlation analysis. The analysis focused on the top 15 taxa that previously showed significant differences between Wistar and WKY rats. These taxa were correlated with behavioral parameters, including indices from the open field test (OFT: total distance, rearing count, and central distance), forced swim test (FST: immobility time), novelty-suppressed feeding test (NSF: latency to feed), intestinal transit time (ITT), and body weight changes. As shown in the figure, OFT measures (total distance, rearing count, and central distance) were negatively correlated with the abundances of Blautia, unclassified_f__Oscillospiraceae, Ruminococcus gauvreauii group, and Bacteroides, and positively correlated with UCG-005 and Enterorhabdus. FST immobility time was positively correlated with the abundances of unclassified_f__Oscillospiraceae, Ruminococcus gauvreauii group, and Marvinbryantia and negatively correlated with UCG-005, Staphylococcus, and Enterorhabdus. NSF latency to feed was positively correlated with the abundances of Blautia, Ruminococcus gauvreauii group, Marvinbryantia, and UCG-008, and negatively correlated with UCG-005, unclassified_c__Bacilli, and Enterorhabdus. Regarding gastrointestinal function, ITT was positively correlated with the abundances of norank_f__Muribaculaceae, Ruminococcus gauvreauii group, unclassified_f__Oscillospiraceae, Colidextribacter, Erysipelotrichaceae_UCG-003, and Marvinbryantia and negatively correlated with UCG-005, unclassified_c__Bacilli, and Enterorhabdus. Body weight changes were positively correlated with the abundances of UCG-005, unclassified_c__Bacilli, and Enterorhabdus and negatively correlated with Blautia, unclassified_f__Oscillospiraceae, Marvinbryantia, Bacteroides, and UCG-008. These correlations suggest that UCG-005, Enterorhabdus, and unclassified_c__Bacilli may exert antidepressant effects while improving gastrointestinal function. Conversely, Ruminococcus gauvreauii group, Blautia, and Marvinbryantia may promote depressive behaviors while inhibiting gastrointestinal function.

### 4.5 EA exerts antidepressant effects, restores gastrointestinal function, and repairs colonic epithelial structure

**Figure 5:**
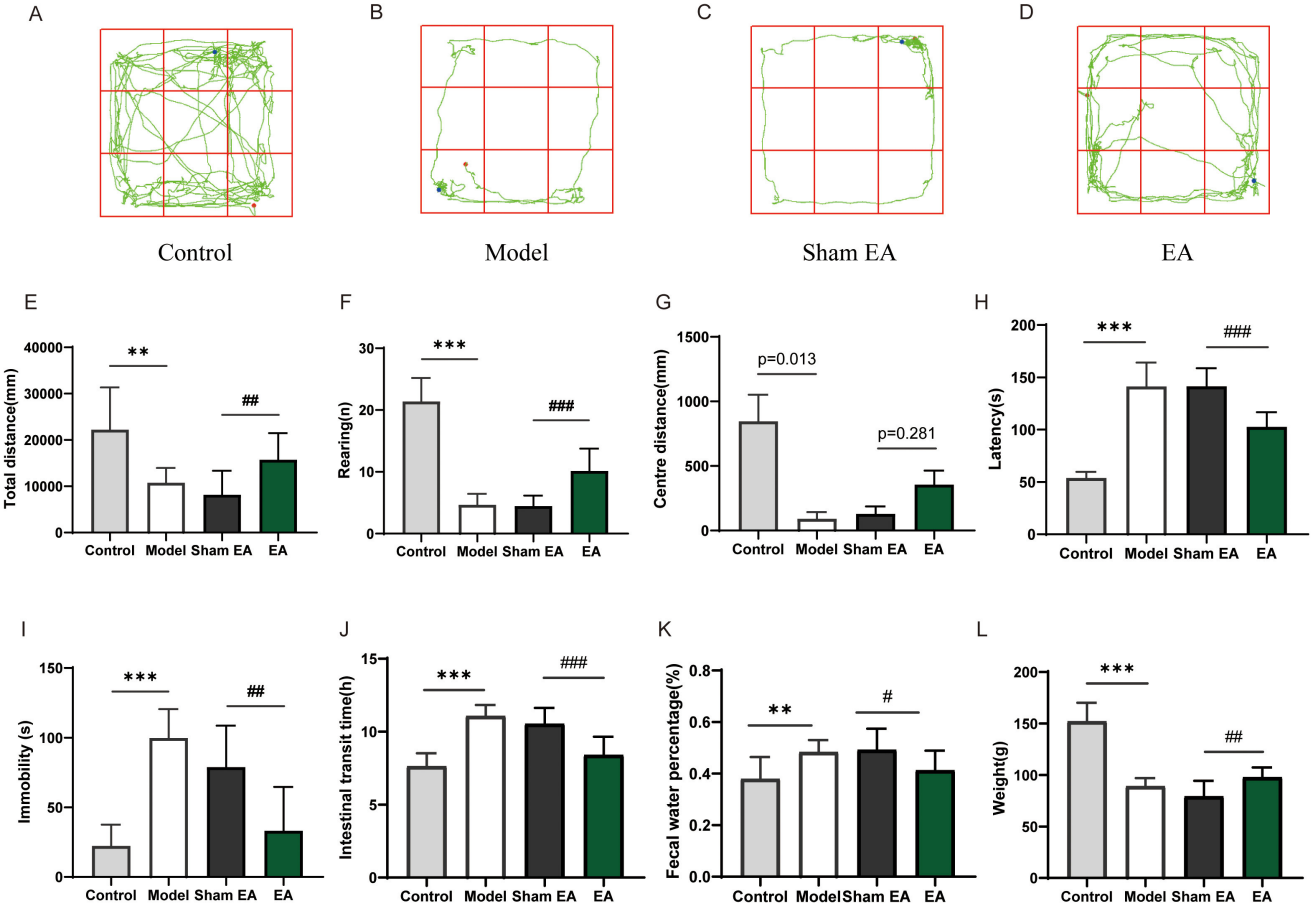
Electroacupuncture can improve depressive-like behaviors and constipation symptoms in depressed rats. **(A–D)** Representative schematic diagrams of motion trajectories recorded during the open field test for each experimental group. **(E)** Quantitative comparison of total moving distance across the different groups in the open field test. **(F)** Quantitative comparison of rearing frequency across the different groups in the open field test. **(G)** Quantitative comparison of central activity distance across the different groups in the open field test. **(H)** Feeding latency assessed in the novelty-suppressed feeding test across the experimental groups. **(I)** Immobility time in the forced swimming test across different groups of rats. **(J)** Comparison of intestinal transit time across different groups of rats. **(K)** Comparison of fecal water content test across different groups of rats. **(L)** Comparison of body weight across different groups of rats. (Control, *n* = 15, Model, *n* = 15, EA, *n* = 15, Sham EA, *n* = 15, Mean ± SD, Control vs. Model *P < 0.05, **P < 0.01, ***P < 0.001; EA vs. Sham EA ^#^*P* < 0.05, ^##^*P* < 0.01, ^###^*P* < 0.001).

Previous studies demonstrated a close association between depression and gut microbiota, particularly involving significant alterations in specific bacterial taxa such as Bacteroidota and Firmicutes, which have been linked to both depressive-like behaviors and gastrointestinal dysfunction in animal models. Given this connection, it is pertinent to explore whether EA can alleviate depressive-like behaviors and constipation symptoms by modulating the gut microbiota. Behavioral test results (Figures 5A–I) revealed that, compared to the control group, the model group exhibited significantly reduced total distance (*F* = 4.584, *df* = 17.42, *P* < 0.01), rearing times (*F* = 15.49, *df* = 19.75, *P* < 0.001), and central distance (*F* = 3.546, *df* = 15.78, *P* < 0.05) in the OFT. In the FST, the model group exhibited longer immobility time (*F* = 11.60, *df* = 25.66, *P* < 0.001), whereas the NSF test indicated an increased latency to feed (*F* = 14.43, *df* = 15.88, *P* < 0.001). Compared to the sham EA group, the EA group showed increased total distance (*F* = 3.786, *df* = 27.71, *P* < 0.01), rearing times (*F* = 5.493, *df* = 19.78, *P* < 0.001), and central distance (*F* = 1.845, *df* = 21.32, *P* = 0.281) in the OFT, although the increase in central distance was not statistically significant. The EA group also exhibited reduced immobility time in the FST (*F* = 4.074, *df* = 27.91, *P* < 0.01) and decreased latency to feed in the NSF (*F* = 6.714, *df* = 26.88, *P* < 0.001). Regarding gastrointestinal function (Figures 5J, K), the model group demonstrated slower weight gain (*F* = 12.31, *df* = 19.19, *P* < 0.001), increased ITT (*F* = 11.32, *df* = 27.37, *P* < 0.001), and higher fecal water content (*F* = 4.197, *df* = 21.76, *P* < 0.01) compared to the control group. Compared to the sham EA group, the EA group showed increased weight (*F* = 4.052, *df* = 23.50, *P* < 0.01), reduced ITT (*F* = 4.950, *df* = 27.48, *P* < 0.001), and decreased fecal water content (*F* = 2.767, *df* = 27.85, *P* < 0.05). These results demonstrate that EA can effectively improve depressive-like behaviors and constipation symptoms in depressed rats.

**Figure 6:**
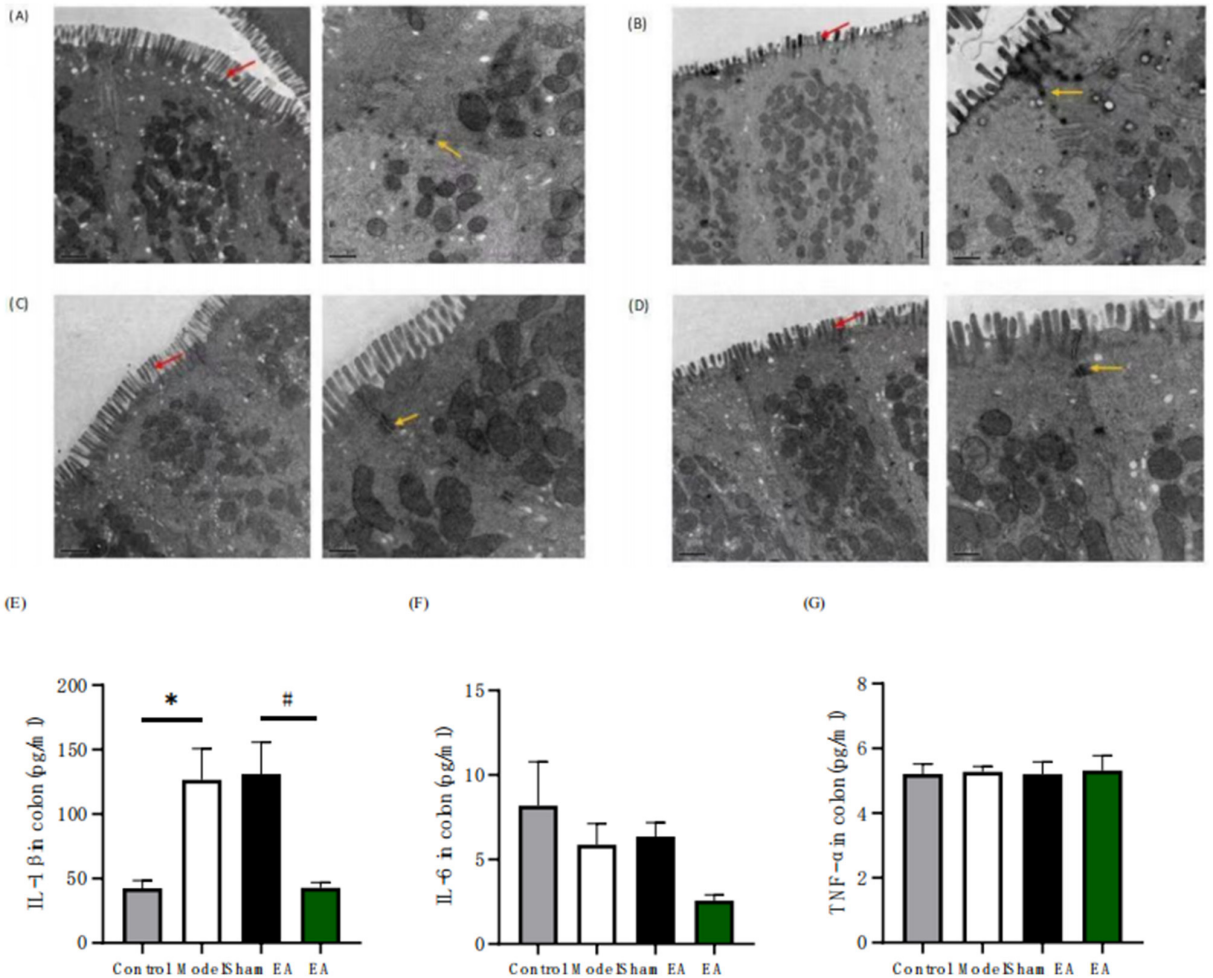
Electroacupuncture effectively promotes the restoration of colonic epithelial ultrastructure in depressed-constipated rats. **(A)** Wistar rat colonic ultrastructure. **(B)** WKY rat colonic ultrastructure. **(C)** EA group colonic ultrastructure. **(D)** Sham EA colonic ultrastructure. (Red arrows indicate microvilli, while yellow arrows denote tight junction proteins. Left panel: 10,000 × magnification; Right panel: 20,000 × magnification). **(E)** Concentration of IL-1β in the colon. **(F)** Concentration of IL-6 the colon. **(G)** Concentration of TNF-α in the colon.

To further evaluate the therapeutic effects of EA on the intestinal microenvironment, we assessed colonic tissue ultrastructure using transmission electron microscopy (TEM) across four experimental groups (Figures 6A–D). Colonic tissues from Wistar rats showed intact architecture with uniformly aligned microvilli of consistent length and density. In contrast, WKY rats exhibited pronounced ultrastructural disruptions, including disorganized microvilli arrangement, significantly shortened microvilli, and diminished expression of tight junction proteins. EA-treated WKY rats demonstrated notable improvements in colonic ultrastructure, characterized by restored microvilli organization, increased microvillus length, and enhanced tight junction protein expression. Meanwhile, the proinflammatory cytokine IL-1β was significantly elevated in the colon of WKY ratsx (*F* = 3, *df* = 8.025, *P* = 0.0017), an effect that was effectively reversed by EA treatment (Figure 6E). Furthermore, we noted that the levels of other proinflammatory cytokines, IL-6 (*F* = 3, *df* = 2.407, *P* = 0.105) and TNF-α (*F* = 3, *df* = 0.0248, *P* = 0.9945), showed no significant differences between Wistar and WKY rats (Figures 6F, G). These results collectively suggest that electroacupuncture can ameliorate intestinal inflammation and protect the intestinal barrier function.

### 4.6 EA effectively balances gut microbiota homeostasis in depressed-constipated rats

**Figure 7:**
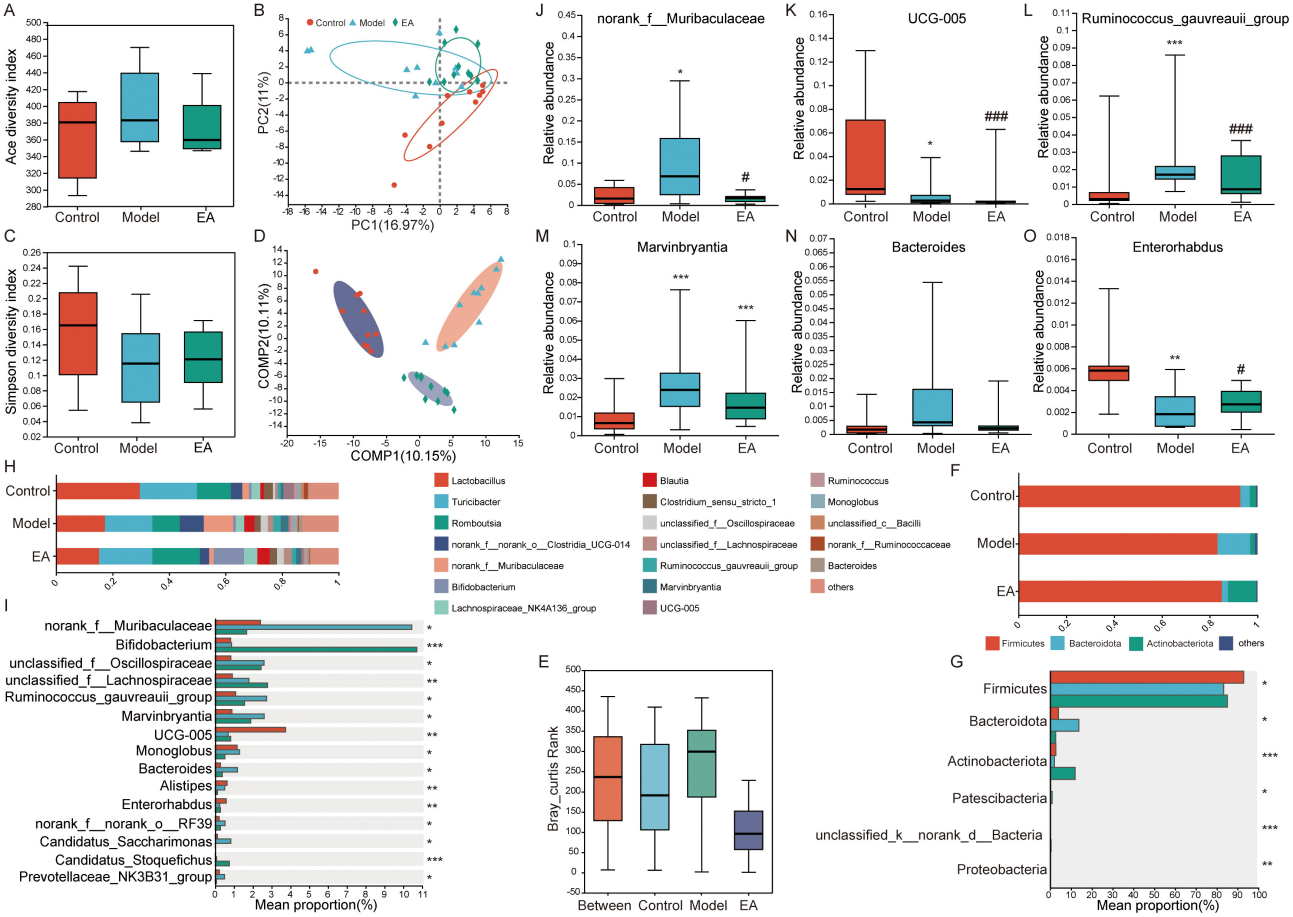
Electroacupuncture effectively balances gut microbiota homeostasis in depressed-constipated rats. **(A)** Ace index of α-diversity in the control, model, and electroacupuncture groups. **(B)** PCA analysis across the three groups. **(C)** Simpson index of α-diversity in the control, model, and electroacupuncture groups. **(D)** PLS-DA analysis across groups. **(E)** Beta diversity based on Bray-Curtis rank across the control, model, and electroacupuncture groups. **(F)** Composition of intestinal flora at the phylum level in the control, model, and electroacupuncture groups. **(G)** Differential analysis of gut flora across three groups at the phylum level. **(H)** Composition of intestinal flora at the genus level in the control, model, and electroacupuncture groups. **(I)** Differential analysis of gut flora across three groups at the genus level. **(J–O)** Relative abundances of six genera showing significant differences between both the control and model groups, as well as between the model and EA groups. (Control, *n* = 10, Model, *n* = 10, EA, *n* = 10, Kruskal-Wallis rank sum test was used for multi-group comparison and *post-hoc, t* test was used to compare the two groups. Control vs. Model *P < 0.05, **P < 0.01, ***P < 0.001; Model vs. EA ^#^*P* < 0.05, ^##^*P* < 0.01, ^###^*P* < 0.001).

To better understand whether the antidepressant effects of EA involve modulation of the microbiota–gut–brain axis, 16S rRNA gene sequencing was performed on fecal samples collected after EA treatment (Figures 7A–O). Alpha diversity analysis was conducted to assess microbial richness and diversity among the three experimental groups. Consistent with previous findings, the model group showed alterations in microbial diversity relative to controls. Although there were no statistically significant differences in the Ace and Simpson indices between the model and EA groups, the Ace index was slightly lower in the EA group, suggesting a reduction in species richness following EA treatment. Beta diversity analysis based on weighted Bray-Curtis distances reflected the similarity in microbial community composition across the three groups, showing significant structural differences between the groups. Additionally, PLS-DA further confirmed these group-specific differences. To better understand the microbial shifts, we compared taxa at both the phylum and genus levels.

At the phylum level, Firmicutes, Bacteroidota, and Actinobacteriota were dominant in all three groups. Compared to the control group, the model group exhibited a significant decrease in the relative abundance of Firmicutes and Actinobacteriota, along with a significant increase in Bacteroidota. Notably, EA treatment resulted in a significant increase in Actinobacteriota and a reduction in Bacteroidota compared to the model group. At the genus level, the predominant genera included Lactobacillus, Turicibacter, Romboutsia, norank_f__norank_o__Clostridia_UCG-014, norank_f__Muribaculaceae, Bifidobacterium, Lachnospiraceae_NK4A136_group, Blautia, Clostridium_sensu_stricto_1, unclassified_f__Oscillospiraceae, unclassified_f__Lachnospiraceae, Ruminococcus gauvreauii group, Marvinbryantia, UCG-005, Ruminococcus, Monoglobus, unclassified_c__Bacilli, norank_f__Ruminococcaceae, and Bacteroides. Compared to the control group, the model group exhibited a significant increase in the abundance of genera such as norank_f__Muribaculaceae, unclassified_f__Oscillospiraceae, unclassified_f__Lachnospiraceae, Ruminococcus gauvreauii group, Marvinbryantia, and Bacteroides and a significant decrease in the abundance of UCG-005, Alistipes, and Enterorhabdus. Compared to the model group, the EA group showed an increase in the abundance of genera such as Bifidobacterium and unclassified_f__Lachnospiraceae and a marked decrease in the abundance of genera such as norank_f__Muribaculaceae, Ruminococcus gauvreauii group, Marvinbryantia, Monoglobus, Bacteroides, norank_f__norank_o__RF39, and Candidatus_Saccharimonas.

Finally, we listed the top ten significant difference microbiota of Wistar and WKY, and marked the influence of FMT and EA on them, as well as the influence on behavior (Table 1). (Where there are differences, they are indicated by arrows, and those without differences are indicated by-).

**Table 1:** Comparison of FMT and EA interventions on gut microbiota and behavioral outcomes.

**Behavior**	**FMT**	**EA**	**Gut Microbiota**	**FMT**	**EA**
Total distance	–	↑	norank_f__Muribaculaceae	–	↓
Rearing	↑	↑	g__Blautia	–	–
Center distance	↑	–	UCG-005	↑	↑
Immobility	↓	↓	Ruminococcus_gauvreauii_group	↓	↓
Latency	↓	↓	Marvinbryantia	–	↓
Intestinal transit time	↓	↓	unclassified_f__Oscillospiraceae	–	–
Weight	–	↑	Bacteroides	–	–
			Enterorhabdus	–	↑
			norank_f__norank_o__RF39	–	↓
			g__Colidextribacter	↑	–

## 5 Discussion

**Figure 8:**
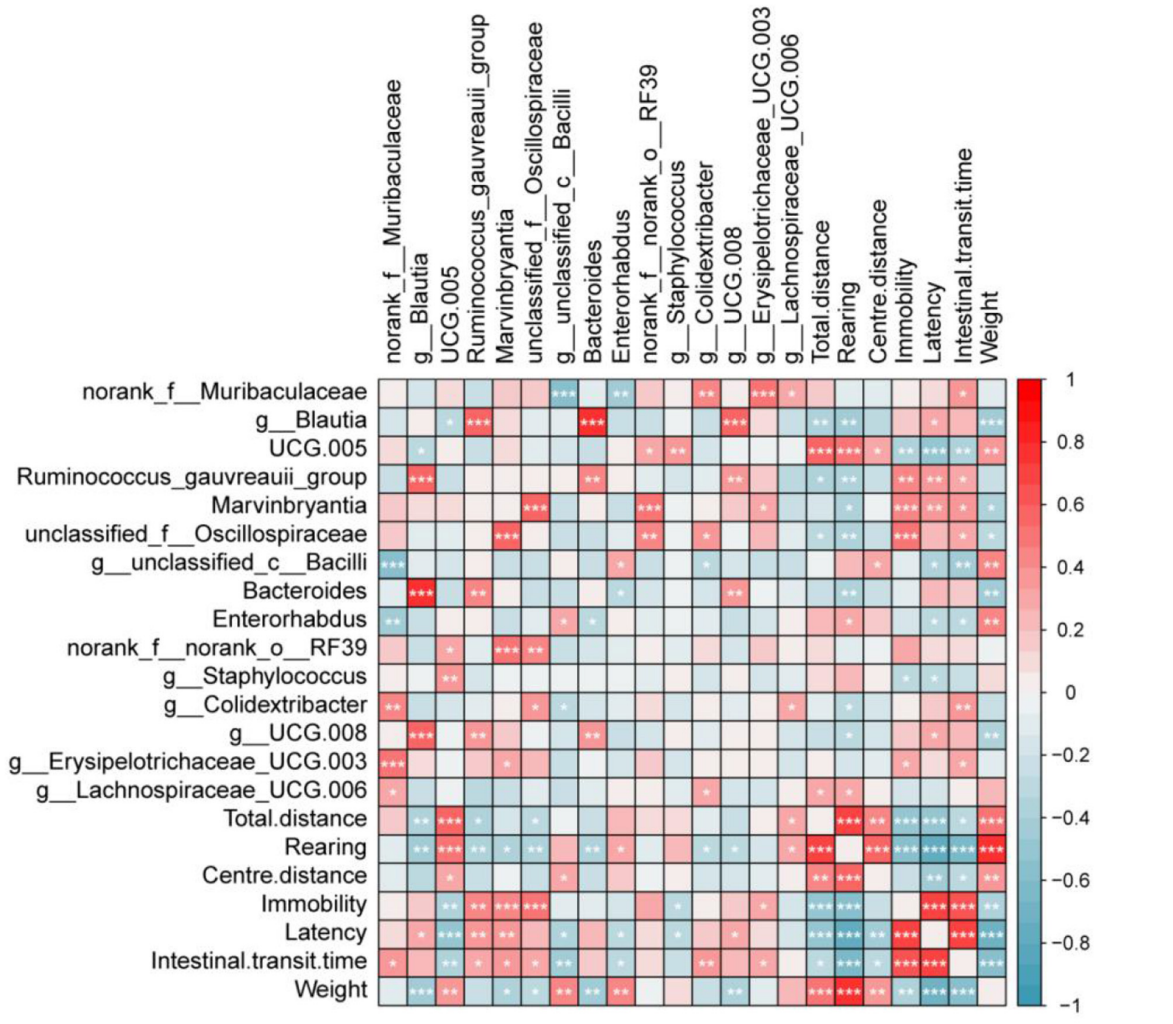
Correlation analysis between depressive behaviors and gut microbiota in rats. Red means positive correlation and green means negative correlation, *P < 0.05, **P < 0.01, ***P < 0.001.

In this study, WKY rats were used to establish a model of depression, and compared to Wistar rats serving as controls, they exhibited marked depressive-like behaviors accompanied by impaired gastrointestinal function (Figure 1). Comparative analysis of gut microbiota revealed distinct microbial signatures, identifying several taxa—UCG-005, Enterorhabdus, unclassified_c__Bacilli, Ruminococcus gauvreauii group, Blautia, and Marvinbryantia—that may jointly influence both depressive-like behaviors and gastrointestinal function (Figure 8). The functional relevance of these microbial candidates was further confirmed through FMT experiments (Figures 3, 4). Moreover, EA treatment alleviated both depressive behaviors and gastrointestinal dysfunction while modulating microbiota abundance (Figure 5).

The co-occurrence of depressive behaviors and constipation symptoms in WKY rats may be associated with abnormal expression of intestinal VIP and ghrelin. VIP slows gastrointestinal transit by reducing intestinal smooth muscle contraction frequency (Zhu et al., 2020; Jia et al., 2024), while ghrelin promotes gastrointestinal propulsion through vagus nerve activation (Shao et al., 2022). Dysregulation of both peptides has been documented in patients with irritable bowel syndrome (IBS) (Furgała et al., 2023). Both VIP and ghrelin are implicated in depression pathology: reduced ghrelin levels are common in depressed patients (Barim et al., 2009), its genetic polymorphism is associated with major depressive disorder (Nakashima et al., 2008), and exogenous ghrelin exhibits antidepressant effects (Lutter et al., 2008). VIP dually regulates intestinal motility and emotional behavior (Huang et al., 2007) and mediates stress-induced gastrointestinal disturbances (Shen et al., 2006). Although ghrelin receptor agonists enhance intestinal neuronal sensitivity to GLP-1 signaling (Buckley et al., 2019), our research data highlight VIP and ghrelin -dominant regulation as the pivotal mechanism for simultaneous alleviation of depressive and gastrointestinal symptoms.

In recent years, gut dysbiosis has been increasingly recognized as a central factor influencing metabolic function, GI physiology, and neurobehavioral outcomes simultaneously (De Palma et al., 2017; Bruce-Keller et al., 2015; Flint et al., 2012). Analysis of the gut microbiota in WKY rats—presenting with both depressive-like behavior and constipation—revealed significant differences in microbial composition compared to control Wistar rats. Notably, alpha diversity was increased, beta diversity was altered, and distinct shifts in microbial composition were observed. At the phylum level, WKY rats showed increased relative abundances of Proteobacteria, Bacteroidota, and Desulfobacterota and a decrease in the relative abundance of Firmicutes. At the genus level, there were notable increases in the relative abundances of norank_f__Muribaculaceae, Blautia, Ruminococcus gauvreauii group, Marvinbryantia, unclassified_f__Oscillospiraceae, Bacteroides, norank_f__norank_o__RF39, Colidextribacter, UCG-008, and Erysipelotrichaceae_UCG-003 and a decrease in the relative abundances of UCG-005, unclassified_c__Bacilli, Enterorhabdus, Staphylococcus, and Lachnospiraceae_UCG-006.

To further explore whether altered gut microbiota causally contributes to behavioral and gastrointestinal dysfunction, we conducted FMT experiments. Previous studies have demonstrated that FMT can alleviate clinical symptoms in patients with IBS comorbid with anxiety and depression, restore gut microbial homeostasis, and increase the relative abundances of *Bacteroidota* and *Firmicutes* (Guo et al., 2021). FMT has also been reported to improve both gastrointestinal symptoms and microbiota balance in individuals with depression (Kang et al., 2017). In a study by Canakis et al., analysis of microbiota composition before and after FMT revealed significant alterations in *Ruminococcus gnavus, Actinobacteria*, and *Bifidobacterium* at 3 weeks post-treatment, with microbiota profiles of recipients becoming more similar to those of donors by 12 weeks (Canakis et al., 2020). Similarly, Borkent et al. (2022) found that *Ruminococcus* abundance was significantly correlated with the severity of depressive symptoms. In our study, WKY rats that received fecal microbiota from healthy Wistar rats showed significant improvements in both depressive-like behaviors and constipation symptoms. Conversely, Wistar rats that received fecal transplants from WKY rats developed depressive-like behaviors and constipation. Correlation analyses further revealed that these phenotypic changes were closely associated with specific microbial taxa, including *norank_f__Muribaculaceae, Blautia, UCG-005, Ruminococcus gauvreauii group, Marvinbryantia, unclassified_f__Oscillospiraceae, unclassified_c__Bacilli*, and *Enterorhabdus*.

Previous studies have found a negative correlation between Enterorhabdus and risk factors for IBD (Hov et al., 2015). In a mouse model of ulcerative colitis, the relative abundance of Enterorhabdus was observed to decrease, whereas probiotic dietary therapy increased its relative abundance and improved gut function (Liu et al., 2022). Additionally, Enterorhabdus has been linked to genetic variations in the human leukocyte antigen (HLA) complex (Xiang et al., 2021), located on chromosome 6p21. This region contains 252 protein-coding genes, approximately one-third of which are believed to be involved in immune regulation. Variations within the HLA complex are well-established determinants of susceptibility to infectious and inflammatory diseases. In the context of mental health, reduced Enterorhabdus abundance has also been reported in a chronic restraint stress (CRS)-induced depression model, where it was found to be strongly associated with depressive-like behavior and altered kynurenine pathway signaling (Deng et al., 2021). Our findings also suggest a potential pathogenic role for the *Ruminococcus gauvreauii* group in the manifestation of both depressive behavior and gastrointestinal dysfunction. Previous research has reported a significant correlation between *Ruminococcus gauvreauii* and depression scores, as measured by the Depression, Anxiety, and Stress Scales (DASS), with higher abundance observed in mildly to moderately depressed individuals compared to healthy controls (Chahwan et al., 2019). Similarly, in a chronic unpredictable mild stress (CUMS)-induced depression model, an elevated abundance of this genus was observed, which was subsequently reversed following FMT that ameliorated depressive-like behavior (Cai et al., 2022). Interestingly, norank_f__Muribaculaceae, typically considered beneficial bacteria that can alleviate intestinal mucosal inflammation in mice (Wang et al., 2016), showed an increase in WKY rats. This family of bacteria has been widely associated with improvements in ulcerative colitis in numerous studies (Liu et al., 2021; Wu et al., 2023). However, our study observed an increased abundance of norank_f__Muribaculaceae in WKY rats. This could be related to the specific comorbid condition of depression with constipation, but further investigation into the specific functions of norank_f__Muribaculaceae is needed to clarify these results.

Clinical studies have demonstrated that acupuncture can significantly alleviate emotional symptoms in patients with depression while concurrently improving gastrointestinal function, which is closely associated with modulation of the gut microbiota (Zhou et al., 2023; Yan et al., 2023). Our study observed that EA may improve depressive-like behaviors and alleviate constipation in WKY rats by modulating the gut microbiota, while we also identified that EA treatment significantly improved colonic ultrastructure in rats, characterized by more orderly arranged microvilli, significantly elongated microvillus length, indicating restored intestinal barrier function. The underlying mechanism may be achieved through the regulation of the gut microbiota-metabolism-intestinal barrier-inflammation axis. Consistent with our findings, numerous studies have demonstrated that EA treatment can remodel the gut microbiota structure by increasing the abundance of beneficial bacteria and reducing pro-inflammatory bacteria, while simultaneously promoting the production of microbial metabolites such as short-chain fatty acids (SCFAs) (Duan et al., 2024). These changes contribute to the restoration of intestinal barrier function and ameliorated intestinal inflammation (Yang et al., 2023; Wang et al., 2020). Emerging evidence indicates that intestinal inflammation is closely associated with the development of neuroinflammation (White et al., 2025) which may constitute a shared mechanism through which EA simultaneously ameliorates both depression and constipation via microbiota-mediated pathways.

## 6 Limitations and future directions

While this study employed 16S rRNA sequencing to analyze gut microbiota composition, this approach has inherent methodological constraints. Although effective for taxonomic classification, 16S sequencing cannot resolve functional differences between closely related bacterial strains (e.g., metabolic activity variations among short-chain fatty acid-producing bacteria) and lacks direct functional gene profiling. Future investigations should integrate shotgun metagenomics and metabolomics (e.g., targeted quantification of short-chain fatty acids and tryptophan metabolites) to comprehensively elucidate the microbiota-metabolism-brain behavior regulatory cascade. The gut microbiota influences the gut-brain axis through microbial metabolites (e.g., butyrate, 5-HT precursors) and immunomodulation (e.g., LPS-induced inflammation). Additionally, while placebo effects of EA were partially controlled via sham acupuncture, more rigorous designs—such as non-contact laser acupuncture—should be implemented to enhance specificity.

## 7 Conclusion

**Figure 9:**
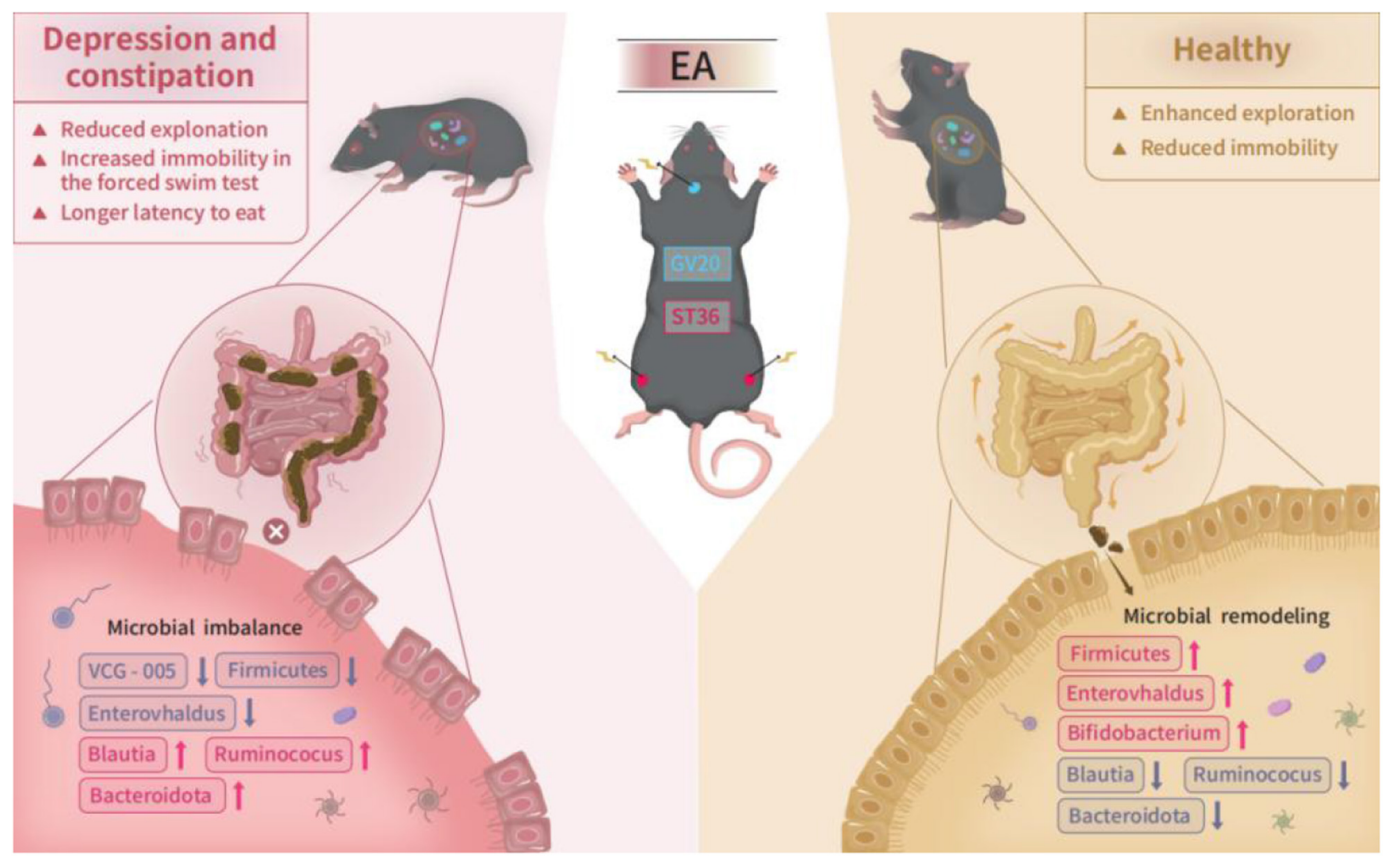
Schematic diagram illustrating the proposed mechanism by which EA alleviates depression with constipation via gut microbiota modulation in WKY rats.

This study demonstrated that WKY rats manifest both depressive-like behaviors and constipation symptoms and identified specific gut microbiota associated with these phenotypes. Through fecal microbiota transplantation (FMT), we established a causal relationship between altered microbiota and the observed behavioral and gastrointestinal dysfunctions. Additionally, EA was shown to simultaneously alleviate these symptoms, potentially through modulation of the gut microbiota. While our findings highlight several key bacterial genera involved in this process (Figure 9).

## Data Availability

The datasets presented in this study can be found in online repositories. The names of the repository/repositories and accession number(s) can be found in the article/supplementary material.
